# Berberine Attenuates Cerebral Ischemia-Reperfusion Injury Induced Neuronal Apoptosis by Down-Regulating the CNPY2 Signaling Pathway

**DOI:** 10.3389/fphar.2021.609693

**Published:** 2021-04-28

**Authors:** Lina Zhao, Huanming Li, Qian Gao, Jin Xu, Yongjie Zhu, Meili Zhai, Peijun Zhang, Na Shen, Yanbo Di, Jinhui Wang, Tie Chen, Meina Huang, Jinglai Sun, Chong Liu

**Affiliations:** ^1^ Department of Anaesthesiology, Tianjin Hospital, Tianjin, China; ^2^ Department of Cardiology, Tianjin 4th Centre Hospital, The Fourth Central Hospital Affiliated to Nankai University, The Fourth Center Clinical College of Tianjin Medical University, Tianjin, China; ^3^ Department of Emergency Medicine, Tianjin 4th Centre Hospital, The Fourth Central Hospital Affiliated to Nankai University, Tianjin, China; ^4^ Department of Pathology, First People’s Hospital of Aksu, Xinjiang, China; ^5^ Department of Anaesthesiology, Tianjin Central Hospital of Gynecology Obstetrics, Gynecology Obstetrics Hospital of Nankai University, Tianjin, China; ^6^ Department of Central Laboratory, Tianjin 4th Centre Hospital, The Fourth Central Hospital Affiliated to Nankai University, Tianjin, China; ^7^ Department of Anaesthesiology, Tianjin 4th Centre Hospital, The Fourth Central Hospital Affiliated to Nankai University, The Fourth Center Clinical College of Tianjin Medical University, Tianjin, China; ^8^ Department of Anaesthesiology, Wuqing People’s Hospital, Tianjin, China; ^9^ Department of Biomedical Engineering, Tianjin Key Laboratory of Biomedical Detecting Techniques and Instruments, Tianjin University, Tianjin, China

**Keywords:** berberine, cerebral ischemia-reperfusion injury, apoptosis, CNPY2, pathway

## Abstract

Berberine (BBR) has a neuroprotective effect against ischemic stroke, but its specific protective mechanism has not been clearly elaborated. This study explored the effect of BBR on the canopy FGF signaling regulator 2 (CNPY2) signaling pathway in the ischemic penumbra of rats. The model of cerebral ischemia-reperfusion injury (CIRI) was established by the thread embolization method, and BBR was gastrically perfused for 48 h or 24 h before operation and 6 h after operation. The rats were randomly divided into four groups: the Sham group, BBR group, CIRI group, and CIRI + BBR group. After 2 h of ischemia, followed by 24 h of reperfusion, we confirmed the neurologic dysfunction and apoptosis induced by CIRI in rats (*p* < 0.05). In the ischemic penumbra, the expression levels of CNPY2-regulated endoplasmic reticulum stress-induced apoptosis proteins (CNPY2, glucose-regulated protein 78 (GRP78), double-stranded RNA-activated protein kinase-like ER kinase (PERK), C/EBP homologous protein (CHOP), and Caspase-3) were significantly increased, but these levels were decreased after BBR treatment (*p* < 0.05). To further verify the inhibitory effect of BBR on CIRI-induced neuronal apoptosis, we added an endoplasmic reticulum-specific agonist and a PERK inhibitor to the treatment. BBR was shown to significantly inhibit the expression of apoptotic proteins induced by endoplasmic reticulum stress agonist, while the PERK inhibitor partially reversed the ability of BBR to inhibit apoptotic protein (*p* < 0.05). These results confirm that berberine may inhibit CIRI-induced neuronal apoptosis by downregulating the CNPY2 signaling pathway, thereby exerting a neuroprotective effect.

## Introduction

Ischemic stroke, also known as cerebral infarction, is a common and frequent clinical condition (Global Burden of Disease Study, 2015). It is mainly caused by the blockage of cerebral blood vessels, which leads to the dysfunction of regional cerebral blood supply. Its disability rate, recurrence rate and mortality rate are high, seriously endangering human physical and mental health (Poustchi et al., 2021). The progression of clinical and basic research has led to improvements in the treatment of stroke, in particular mechanical thrombectomy and thrombolysis. However, the narrow treatment time window and subsequent complications remain difficult problems for clinicians (Burrows et al., 2015). Among them, severe brain tissue injury may occur after vascular recanalization; this type of damage is called cerebral ischemia-reperfusion injury (CIRI) (Sun et al., 2018) and is an important cause of subsequent complications, which poses a new challenge to researchers. The discovery of methods to reduce CIRI is an area of active research, and elucidating the mechanism of CIRI can provide new ideas and targets for treatment.

With the national investment in Chinese medical research, the unique curative effect of traditional Chinese medicine for chronic diseases has been confirmed (Qiao et al., 2020). There are many kinds of traditional Chinese medicine (TCM). TCM has no obvious toxicity or side effects and has comprehensive therapeutic effects that cannot be replaced by Western medicine. In recent years, many TCMs have shown promise for the clinical diagnosis and treatment of CIRI, and new theories have also been proposed to continuously expand and improve the understanding of TCM mechanisms (Huang et al., 2019; Long et al., 2020). As a common clinical Chinese medicine, berberine (BBR) is widely used as an antipyretic analgesic and as an antibacterial agent for intestinal infections (Song et al., 2020). Recent studies have also shown that BBR not only has antioxidant, anti-stress, anti-inflammatory, hypolipidemic and anticancer pharmacological activities, it also has a clear therapeutic role in cardiovascular diseases (Song et al., 2020). Importantly, BBR can play a direct neuroprotective role in cerebral ischemia injury because it can cross the blood-brain barrier (Liu et al., 2019). Recent research has shown, BBR may reduce CIRI-induced neuronal apoptosis by reducing the expression of caspase-3 and caspase-9, increasing the ratio of B lymphocyte tumor-2 (Bcl-2)/Bcl-2 related x protein (Bax) (Zhang et al., 2016b). Similarly, studies have also found that BBR also alleviated ischemic brain injury by reducing intracellular reactive oxygen species (ROS) level and subsequently inhibiting mitochondrial apoptosis pathway (Zhou et al., 2008). Its protective mechanism may involve an extremely complex pathophysiological process, and its specific pharmacological mechanism has not been fully clarified; thus, it is the topic of our in-depth study.

CIRI is an important issue in the mechanism of ischemic cerebrovascular disease injury and is an intensely studied research area in China and abroad. At present, a wide range of scholars believe that insufficient blood flow in local areas of the brain causes a series of complex reactions, such as inflammation, oxidative stress, apoptosis, intracellular calcium overload, and excitatory amino acid toxicity (Datta et al., 2020). These factors interact and influence each other to form a complex regulatory network, leading to neuronal apoptosis/death. CIRI-induced apoptosis is an important route of nerve cell death, which clearly exacerbates neurological deficits in patients with cerebral infarction. Therefore, apoptosis plays a crucial role in the pathological process of CIRI (Uzdensky, 2019). Current research suggests that there are three main signal transduction pathways that regulate apoptosis, namely, the mitochondrial pathway, the death receptor pathway and the endoplasmic reticulum stress (ERS) pathway (Ten and Galkin, 2019; Zhai et al., 2019; Datta et al., 2020). Although the most recent studies have identified the important role of the ERS-mediated apoptosis pathway in CIRI, it is not completely clear how ERS is triggered. Recent studies have found that canopy 2 (canopy FGF signaling regulator 2, CNPY2) is involved in the occurrence of ERS and is the main trigger of the PERK-CHOP pathway (Hong et al., 2017). During the transition of the ER from a nonstressed state to a stressed state, the CNPY2 binding partner switches from GRP78 to PERK, thereby selectively activating the PERK-CHOP-mediated apoptosis signaling pathway (Urra and Hetz, 2017). However, our understanding of the role of CNPY2 is limited, and its mechanism in CIRI requires further study.

Based on the neuroprotection mechanism of BBR and the important role of ERS apoptosis pathway in CIRI, this study established a CIRI rat model to further explore the role and mechanism of ERS pathway in BBR neuroprotection. The results reveal part of the neuroprotective mechanism of BBR at the molecular biological level and provide a theoretical basis for its clinical application in the treatment of ischemic stroke.

## Materials and Methods

### Animals

Healthy male Sprague Dawley (SD) rats, approximately 8–10 weeks old, weighing 210–260 g, were provided by the Beijing Charles River Experimental Animal Technology Co., Ltd. The feeding environment was well ventilated during the experiment. The room temperature was 22–25°C, the humidity was 40–70%, and the circadian rhythm was maintained at 12 h/12 h. All animals had free access to food and water. All animal experiments were approved by the Experimental Animals Ethics Committee of the Tianjin 4th Centre Hospital (The Fourth Central Hospital Affiliated to Nankai University) and the Experimental Animal Ethics Committee of Institute of Radiation Medicine, Chinese Academy of Medical Sciences. All animal programmes are implemented in strict accordance with the provisions of the National Regulations on Experimental Animal Ethics. The associated permit number is IRM-DWLL-2019139.

### Main Reagents

BBR (purity >99%) was purchased from Sigma Aldrich; antibodies against CNPY2, PERK, and CHOP were purchased from Proteintech; p-PERK was purchased from Cell Signaling; and GRP78, GAPDH, Calnexin, and Caspase-3 were all purchased from Abcam. The reagent thapsigargin (TG) was purchased from Sigma, and GSK2606414 (GSK) was purchased from MCE. The TUNEL kit was purchased from Roche.

### Drug Treatment and Grouping

SD rats were randomly divided into 4 groups (12 in each group), namely, the sham operation group (Sham group), BBR treatment group (BBR group), rat cerebral ischemia-reperfusion injury model group (CIRI group), and CIRI + BBR treatment group (CIRI + BBR group). The BBR group and CIRI + BBR group were administrated intragastrically with BBR for 48 h, 24 h before operation and 6 h after operation. The dose of BBR (40 mg/kg) was derived from previous research results and is in the range of BBR doses commonly used in clinical practice (Zhang et al., 2016b; Zhang et al., 2016a; Liu et al., 2019). Correspondingly, rats in the Sham and CIRI groups were intragastrically given equal volumes of saline. The rats in the Sham group and BBR group had the same operation as the CIRI group except the middle cerebral artery was not blocked. Rats that died of anesthesia and surgery were excluded. After 24 h of reperfusion, all rats were scored for neurological impairment, and brain tissue samples were collected for further experiments.

To further verify the experimental results, we added the ER agonist TG (intraperitoneal injection with 0.3 mg/kg) and randomly divided the rats into five groups (6 rats per group): the Sham + DMSO group (Sham + D group), CIRI + D group, CIRI + D + BBR group, CIRI + TG group, and CIRI + TG + BBR group. Simultaneously, in another group of experiments, we added GSK (Intraventricular injection with 90 μg), a PERK-specific inhibitor of the ERS apoptosis pathway (Yan et al., 2017; Meng et al., 2018). According to the experimental requirements, the rats were randomly divided the rats into four groups (6 rats per group): the Sham + D group, CIRI + D group, CIRI + D + BBR group, and CIRI + GSK + BBR group. DMSO is a solvent for TG and GSK. TG and GSK were injected intracerebrally at the beginning of reperfusion. Rats in the Sham and CIRI groups were given an equal volume of DMSO solution, and the rest of the operations were the same as before. At the end of the experiment, all rats were screened by neurological deficit scoring, and samples of ischemic penumbra were separated according to a previous method (Huang et al., 2015; Liu C. et al., 2018), and then frozen in liquid nitrogen for Western blot experiments.

### Establish the Cerebral Ischemia-Reperfusion Injury Model

To maximize the approach to the pathogenesis of stroke in humans, the rat CIRI model was established by the thread suppository method reported by Zea-Longa (Longa et al., 1989). Animals were fasted for 12 h and water for 4 h before surgery. Briefly, the rats were anesthetized by intraperitoneal injection of chloral hydrate (300-350 mg/kg), and then fixed on the operating table in the supine position. After shearing and disinfection, a median cervical incision was performed to fully expose the right common carotid artery (CCA), external carotid artery (ECA) and internal carotid artery (ICA). The CCA proximal end and ECA proximal end were ligated with a ligature, and the ICA distal end was clamped by an arterial clip to temporarily block the blood supply to the brain. Then, a small incision was made in CCA with ophthalmic scissors. The monofilament nylon thread was inserted through the incision, the artery clamp was loosened, and the thread bolt was slowly pushed in the internal carotid artery to a slight resistance. The insertion depth was about 18–20 mm, that is, the successful occlusion of the middle cerebral artery. Cover the neck incision with gauze soaked in saline. After 2 h of ischemia, the nylon thread was removed to restore the blood flow of middle cerebral artery, and cerebral reperfusion was maintained for 24 h. The line end of the Sham group stopped advancing within 10 mm. The common carotid artery was ligated with pulling thread, the incision was disinfected locally, and finally the incision was sutured layer by layer. The rats maintained spontaneous breathing during the operation, and the room temperature was maintained at 22 ± 2°C.

### Modified Neurological Severity Score

After 2 h of cerebral ischemia and 24 h of reperfusion, the mNSS was determined for the rats in each group to assess their neurological function before taking samples. Scoring indicators include exercise, sensory, balance and reflex tests. The lowest score is 0 points and the highest score is 18 points. The higher the score is, the more severe the neurological impairment. The specific operation method and scoring standard of the experiment are provided in the references (Lourbopoulos et al., 2008). The assessors did not know the grouping of the rats.

### Terminal Deoxynucleotide Trans-Ferase-Mediated dUTP Notch Terminal Labelling (TUNEL) Staining

After the experimental rat was anesthetized, the thoracic cavity was quickly opened, and a small incision was cut in the left ventricle and right atrium respectively. Normal saline was to flush blood through a lavage catheter inserted into the left ventricle, and 4% paraformaldehyde was used to perfuse the tissue. Then, the brain was quickly removed and fixed in 4% paraformaldehyde solution. After routine dehydration, transparent and paraffin embedding, continuous coronal slices were made. According to the manufacturer's protocol, paraffin-embedded brain sections were fluorescently stained using an *in situ* apoptosis detection kit (POD; Roche Diagnostics Corp., Indianapolis, IN, United States). Under a fluorescence microscope (400×), 4 fields of view of each brain specimen slice were randomly selected, and the number of cells and apoptotic cells in the visual field were calculated. The apoptotic rate was calculated as the number of apoptotic cells/total number of cells × 100%.

### Immunofluorescence Assay

Serial coronal sections of brain samples were made according to the above experimental procedures, and immunofluorescence testing was performed according to the manufacturer's instructions. Briefly, sections were routinely dewaxed and antigen repaired, and CNPY2 primary antibody (1:250 dilution) or GRP78 primary antibody (1:500) was added and incubated at 4°C overnight. After the slices were washed, the second antibody was added (incubated at 37°C for 30 min), and then the nucleus was stained with DAPI (1:100). The distribution of cells positive for CNPY2 or GRP78 was observed under a fluorescence microscope (400×). Four areas of each sample were randomly captured, and the fluorescence intensity was analyzed using ImageJ software.

### Immunohistochemistry

The above experimental steps were followed to make continuous coronal slices of the brain specimens; immunohistochemical staining was then performed according to the manufacturer's instructions. In short, the sections were routinely dewaxed and antigen repaired, and the endogenous peroxidase activity was eliminated. Next, the sections were incubated with CNPY2 primary antibody (1:800 dilution) at 4°C overnight. After rinsing, a secondary antibody was added to the section and incubated at 37°C for 30 min 3,3′-Diaminobenzidine (DAB) was used for color development at room temperature, and samples were counterstained with the dropwise addition of hematoxylin. After mounting, CNPY2-positive cells were observed under an optical microscope at a magnification of 400×. Brown staining was considered to indicate positive expression. Four areas of each sample were randomly captured and analyzed using ImageJ software. The positive rate of immune cells = positive number of immune cells/(number of positive cells + number of negative cells) × 100%.

### Western Blot Experiment

Protein separation and Western blotting were performed according to the steps described in a previous study (Zhao et al., 2019). In short, the rat brain ischemic penumbra tissue was rapidly homogenized, and the cells were then fully lysed and centrifuged at 4°C at 12000x g for 30 min. The collected tissue homogenate supernatant was used for protein concentration determination (BCA method). An equal amount (30 μg) of protein sample was transferred to a nitrocellulose membrane after 10–12% sodium dodecyl sulfate-polyacrylamide gel electrophoresis (SDS-PAGE). The membrane was incubated with 5% skim milk at room temperature for 2 h and then incubated at 4°C overnight with specific monoclonal antibodies. Primary antibodies included CNPY2 (1:500), GRP78 (1:5000), p-PERK (1:1000), CHOP (1:2000), PERK (1:1000), Caspase-3 (1:2000), Calnexin (1:2000) and glyceraldehyde-3-phosphate dehydrogenase (GAPDH, 1:10000). The next day, the membrane was washed at room temperature and incubated with the corresponding secondary antibody (Solarbio) for 2 h. The protein bands were then developed with an enhanced chemiluminescence solution. Image lab software (Bio-Rad, United States) was used to quantitatively analyze the protein bands.

### Statistical Analysis

The data were statistically analyzed using SPSS19.0 software (SPSS Corporation, United States). The data are expressed in the form of mean ± SEM. For comparison between multiple groups, one-way analysis of variance (ANOVA) and Tukey’s postmortem multiple comparison test were used. Statistical significance was defined as *p* < 0.05. All statistical graphics were processed by GraphPad Prism software (version 5.0, GraphPad Software Inc., United States).

## Results

### Berberine Improves Neurological Dysfunction Induced by Cerebral Ischemia-Reperfusion Injury

**FIGURE 1:**
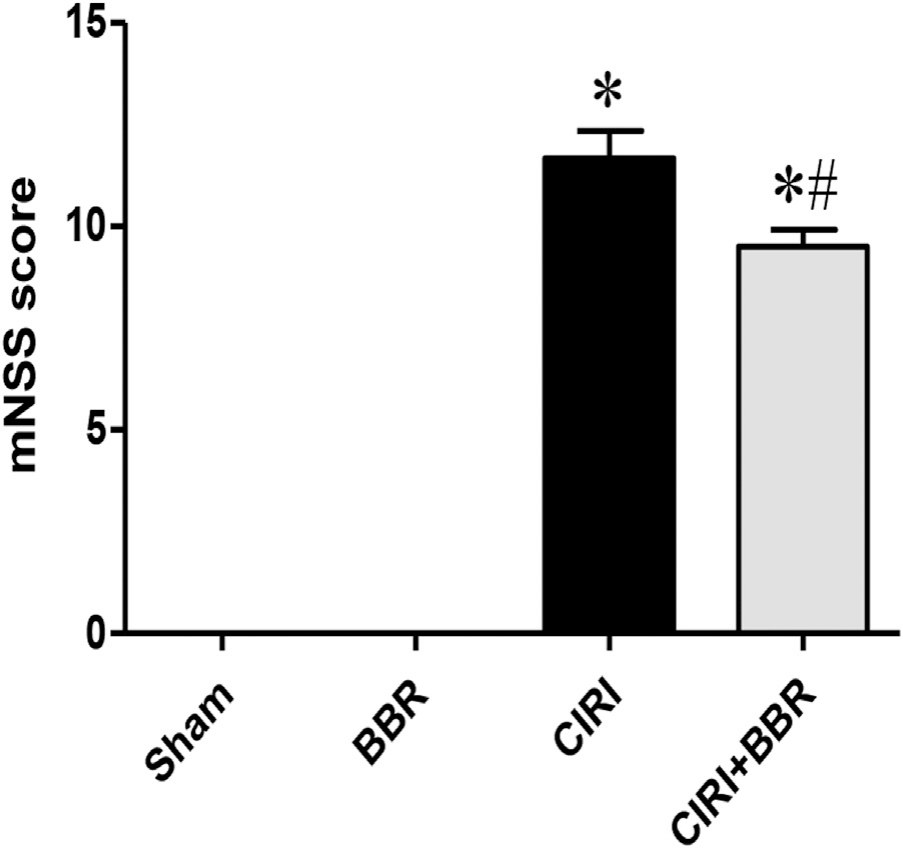
BBR improves neurological dysfunction induced by CIRI. After 24 h of reperfusion injury, neuroethology was evaluated by the Modified Neurological Severity Score (mNSS). Data are shown as the mean ± SEM, *n* = 12 per group. *p < 0.05 compared with Sham group; ^#^ *p* < 0.05 compared with the CIRI group.

After 24 h of reperfusion injury, neuroethology was evaluated by the Modified Neurological Severity Score (mNSS) (Lourbopoulos et al., 2008). There was no statistically significant difference in mNSS score between the Sham group and the BBR group (*p* > 0.05, Figure 1). Compared with that of the Sham group, the neurological function score of the CIRI group was significantly increased (*p* < 0.05). However, the mNSS score of the CIRI + BBR group decreased significantly after 24 h of perfusion, which was statistically significant compared with that of the CIRI group (*p* < 0.05, Figure 1). The mNSS score comprehensively evaluates the neurological deficit after ischemia. The results show that BBR improved the neurological dysfunction caused by CIRI.

### Berberine Attenuates Neuronal Apoptosis in Ischemic Penumbra Induced by Cerebral Ischemia-Reperfusion Injury

**FIGURE 2:**
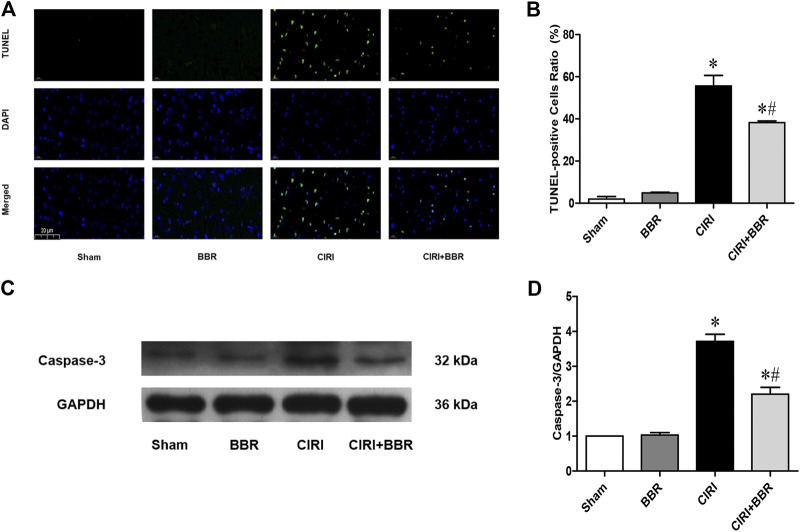
BBR attenuates neuronal apoptosis in ischemic penumbra induced by CIRI. **(A)**, TUNEL staining was performed on slices from cortical ischemic penumbra. **(B)**, Ratio of positive cells. Original magnification is 400×, scale bars is 20 μm. **(C)**, The apoptotic protein cleaved Caspase-3 from cortical ischemic penumbra was determined by Western blot. GAPDH was used as the loading control. *n* = 6. **(D)** Bar graph showing the statistical comparison of Caspase-3 protein levels across groups. Data are shown as the mean ± SEM. *p < 0.05 compared with Sham group; ^#^ *p* < 0.05 compared with the CIRI group.

Twenty-four hours after CIRI, TUNEL staining was used to detect neuron-positive apoptotic cells. The experimental results showed that compared with that in the Sham group, the number of apoptotic cells in the cortical ischemic penumbra of the CIRI group was increased (*p* < 0.05). Compared with that in the CIRI group, the number of positive apoptotic cells in the CIRI + BBR group was significantly reduced (*p* < 0.05) (Figures 2A,B). Western blot analysis showed that Caspase-3 expression in the cortical ischemic penumbra of the CIRI group was significantly higher than that of the Sham group, but after BBR treatment, Caspase-3 expression was significantly decreased (*p* < 0.05, Figures 2C,D). These results show that BBR could inhibit apoptosis and protect neurons.

### Berberine Attenuates Neuronal Apoptosis in Cortical Ischemic Penumbra by Inhibiting Endoplasmic Reticulum Stress Pathway

**FIGURE 3:**
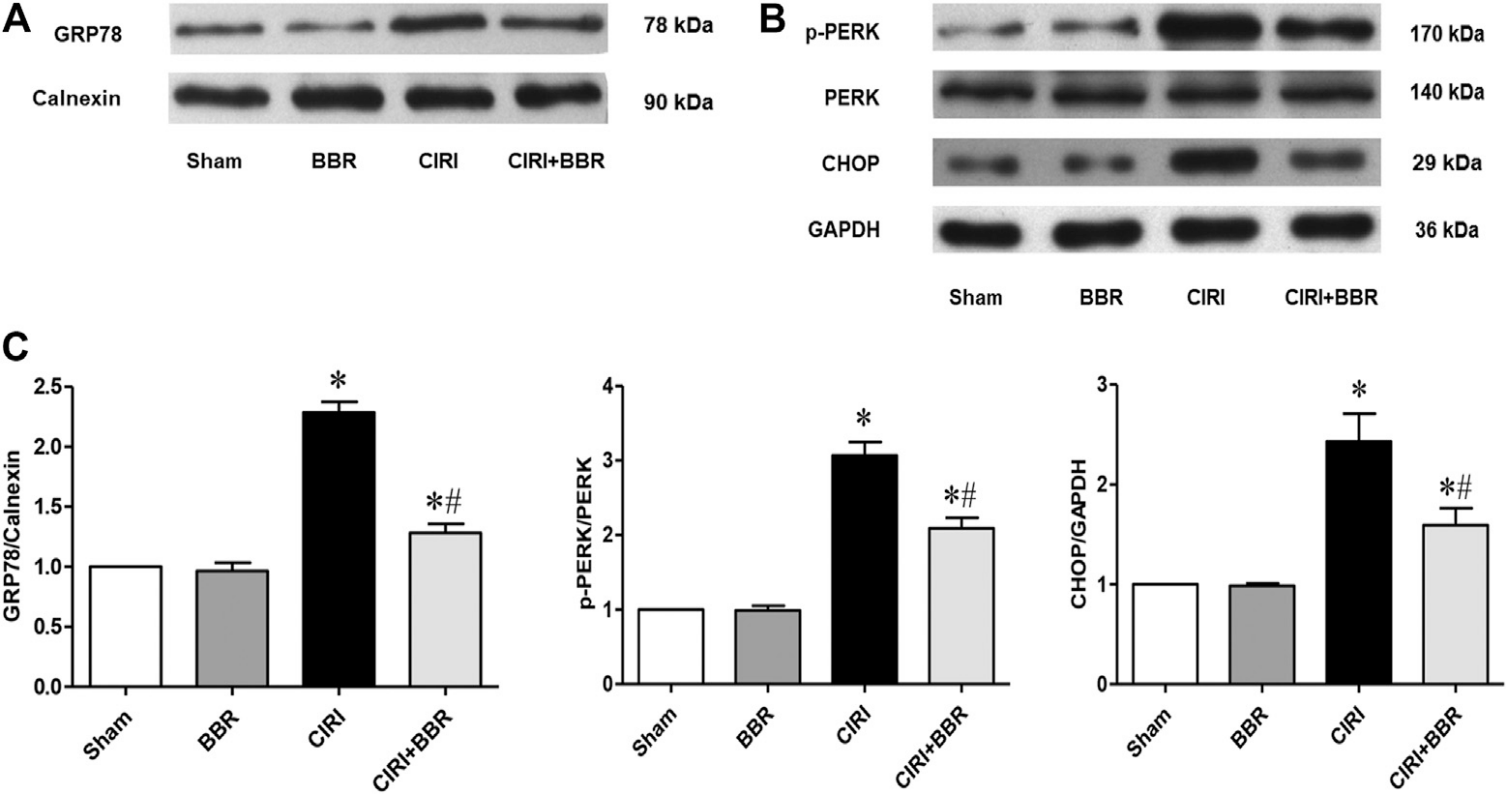
BBR attenuates neuronal apoptosis in cortical ischemic penumbra by inhibiting the ERS pathway. The ERS-related proteins GRP78 **(A)**, p-PERK/PERK and CHOP **(B)** from cortical ischemic penumbra were determined by Western blot. GAPDH was used as the loading control. **(C)**, Bar graph showing the statistical comparison of GRP78, p-PERK/PERK and CHOP protein levels across groups. *n* = 6. Data are shown as the mean ± SEM. *p < 0.05 compared with Sham group; ^#^ *p* < 0.05 compared with the CIRI group.

The ER molecular chaperone protein GRP78 is a marker protein of ERS and is mainly located in the cytoplasm and ER. Under pathological conditions, GRP78 would dissociate from CNPY2, and activate the PERK-CHOP mediated apoptosis signaling pathway, thereby inducing cell apoptosis. After 24 h of CIRI, the expression levels of the ERS apoptosis proteins GRP78, CHOP, and p-PERK/PERK in the ischemic penumbra were detected by Western blot (Figure 3). The results showed that the expression levels of GRP78, CHOP, and p-PERK/PERK were significantly increased in the CIRI group compared with those in the Sham group (*p* < 0.05). However, after BBR treatment, the expression levels of GRP78, CHOP, and p-PERK/PERK were significantly lower than those in CIRI group (*p* < 0.05). These results further suggest that BBR may reduce neuronal apoptosis in the cortical ischemic penumbra by inhibiting the ERS apoptosis pathway.

### Berberine Attenuates Neuronal Apoptosis by Inhibiting CNPY2-Endoplasmic Reticulum Stress in Ischemic Penumbra

**FIGURE 4:**
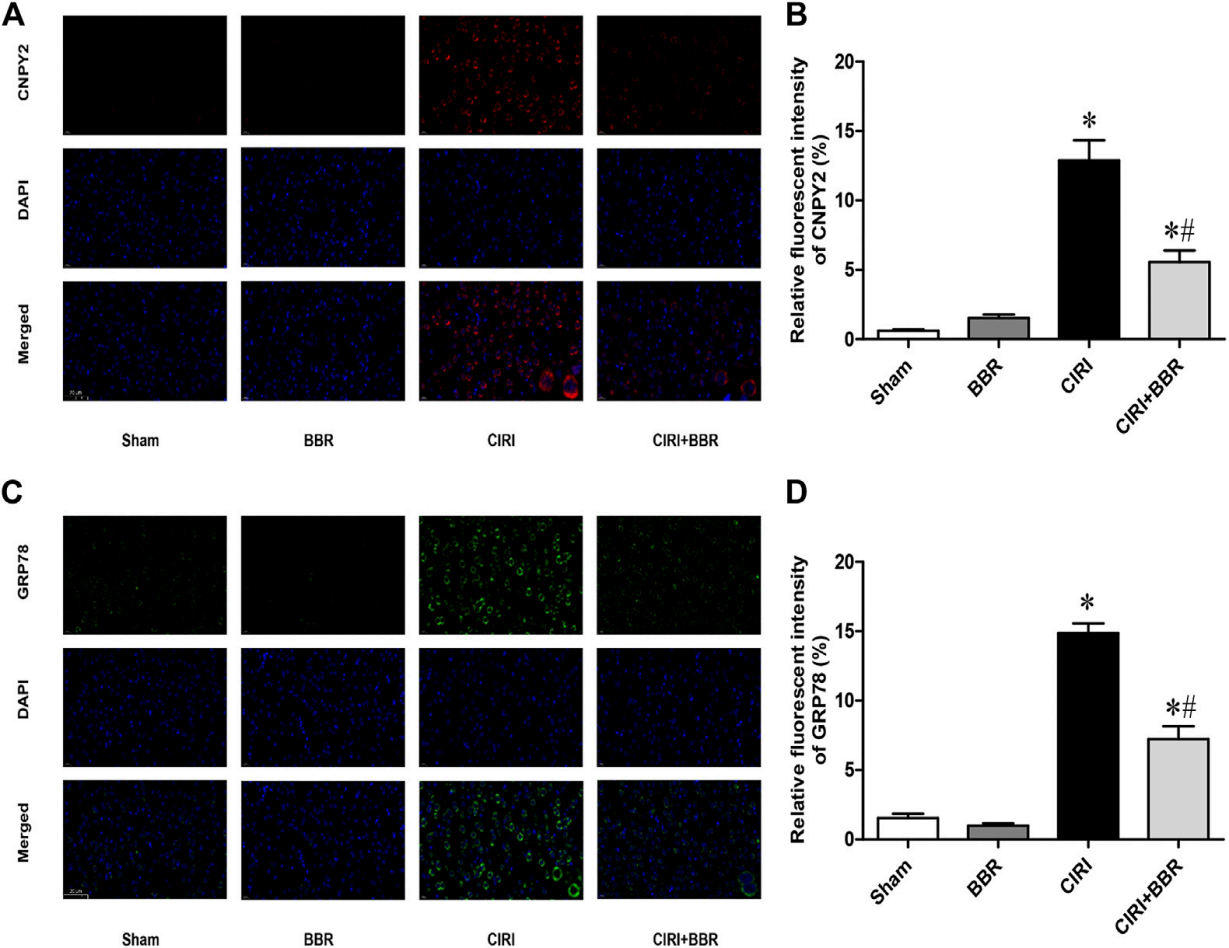
BBR inhibits CNPY2-mediated ERS pathway. The expression of CNPY2 and GRP78 in ischemic brain tissue was analyzed by immunofluorescence staining to further evaluate the effect of BBR on the ERS pathway. **(A)**, The location of CNPY2 in the ischemic penumbra of each group. **(B)**, Ratio of fluorescence intensity of CNPY2 in ischemic penumbra. **(C)**, The location of CNPY2 in the ischemic penumbra of each group. **(D)**, Ratio of fluorescence intensity of CNPY2 in ischemic penumbra. Original magnification is 400×, scale bars is 20 μm *n* = 6. Data are shown as the mean ± SEM. *p < 0.05 compared with the Sham group; ^#^ *p* < 0.05 compared with the CIRI group.

**FIGURE 5:**
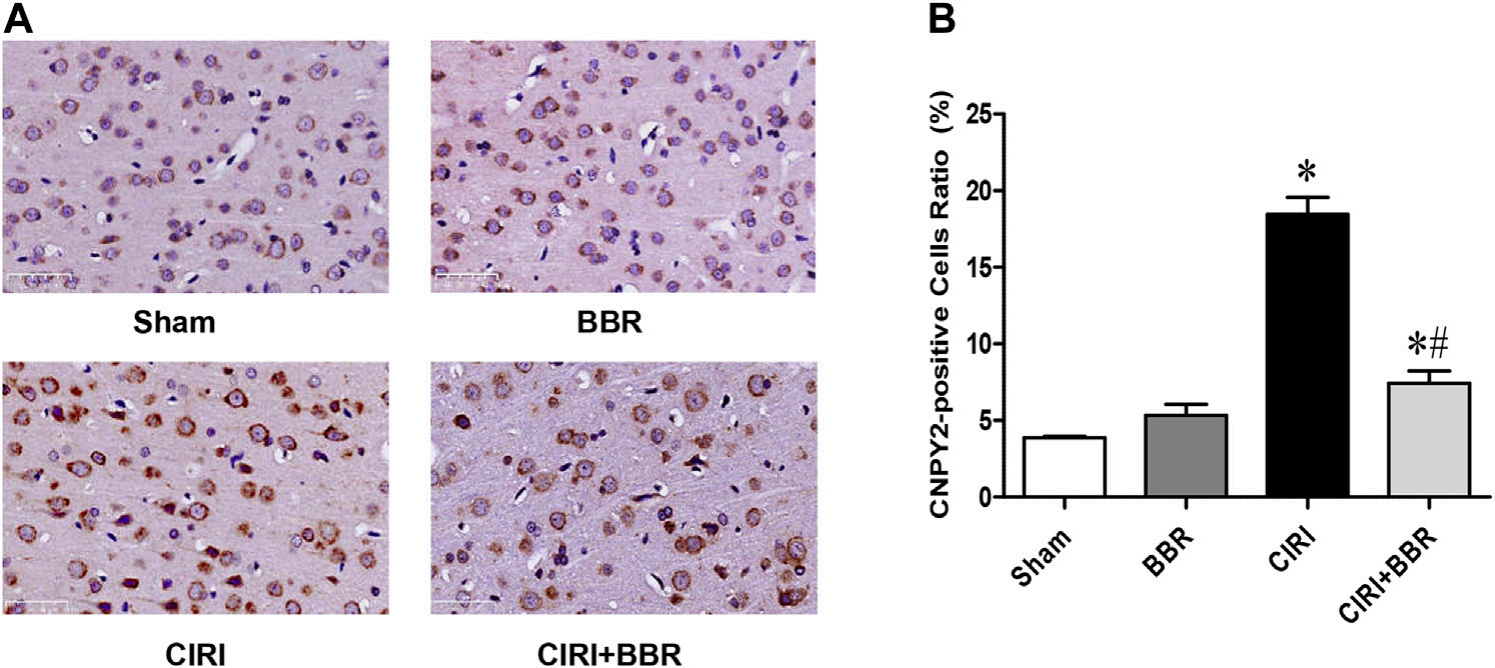
BBR inhibits ERS in cortical ischemic penumbra. **(A)**, Immunohistochemistry results showed that CNPY2-positive neurons were found in the ischemic penumbra area of each model group. **(B)**, Ratio of positive cells. Original magnification is 400×, scale bars is 20 μm *n* = 6. Data are shown as the mean ± SEM. *p < 0.05 compared with Sham group; ^#^ *p* < 0.05 compared with the CIRI group.

**FIGURE 6:**
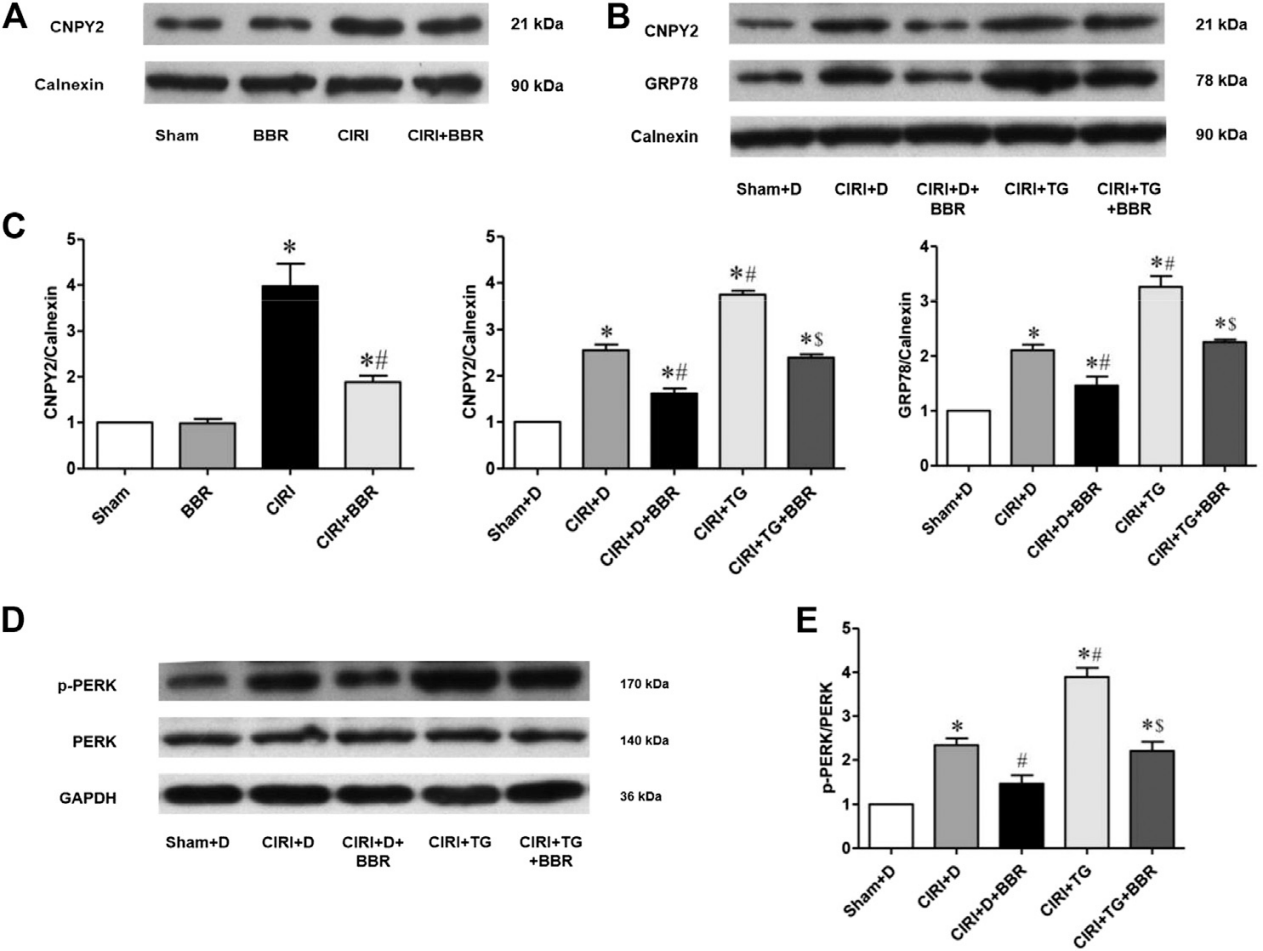
BBR attenuates neuronal apoptosis by inhibiting CNPY2-ERS in ischemic penumbra. The CNPY2-ERS apoptotic signaling proteins CNPY2 **(A,B)**, GRP78 **(B)**, and p-PERK/PERK **(D)**, from cortical ischemic penumbra were determined by Western blot. The results were normalized to the percentage of Calnexin or GAPDH expression. **(C,E)**, Bar graph showing the statistical comparison of CNPY2, GRP78 and p-PERK/PERK protein levels across groups. *n* = 6. Data are shown as the mean ± SEM. *p < 0.05 compared with the Sham group or Sham + D group; ^#^ *p* < 0.05 compared with the CIRI group or CIRI + D group; ^$^ *p* < 0.05 compared with the CIRI + TG group.

CNPY2 is the initiating factor of the ERS signaling pathway and is located in the ER. GRP78 is a marker protein of ERS and is mainly located in the ER and cytoplasm. Their expression levels reflect the degree of ERS activation. CNPY2 and GRP78 expression in ischemic brain tissue was analyzed by immunofluorescence staining to further evaluate the effect of BBR on the ERS pathway. The immunofluorescence results showed that CNPY2 and GRP78 were mainly located in the cytoplasm of the cerebral cortex in the Sham group and showed weak expression (Figure 4). After 24 h of reperfusion, the expression level of CNPY2 in the CIRI group was significantly enhanced, and nuclear translocation occurred; the expression of GRP78 was also significantly enhanced, without obvious nuclear metastasis (*p* < 0.05). After BBR treatment, the expression levels of CNPY2 and GRP78 in the CIRI + BBR group were significantly lower than those in the CIRI group, and the translocation phenomenon was significantly reduced (*p* < 0.05). Similarly, the immunohistochemistry and Western Blot results of CNPY2 in each group were also similar to the immunofluorescence results (Figures 5, 6A). Compared with the Sham group, the expression level of CNPY2 in the ischemic penumbra of the CIRI group was significantly higher (*p* < 0.05). In the CIRI + BBR group, the expression level of CNPY2 was significantly lower than that in the CIRI group (*p* < 0.05). These results suggest that BBR may inhibit apoptosis by regulating the CNPY2-ERS pathway.

To confirm the role of the CNPY2-ERS pathway in CIRI, we used the ER agonist TG to stimulate the occurrence of ERS. Western blotting was used to detect the expression of CNPY2 and ERS apoptotic pathway proteins in ischemic penumbra (Figures 6B,D). The results suggested that compared with those in the Sham + D group, the expression levels of CNPY2, p-PERK/PERK, and GRP78 in the CIRI + TG group were significantly increased (*p* < 0.05). After BBR treatment, the expression levels of CNPY2, p-PERK/PERK, and GRP78 in the CIRI + TG + BBR group were significantly lower than those in the CIRI + TG group (*p* < 0.05, Figures 6C,E). The experimental results showed that BBR may reduce nerve cell apoptosis by inhibiting the CNPY2-ERS pathway in the ischemic penumbra.

### Berberine Attenuates Neuronal Apoptosis by CNPY2-Protein Kinase-Like ER Kinase Pathway in Cortical Ischemic Penumbra

**FIGURE 7:**
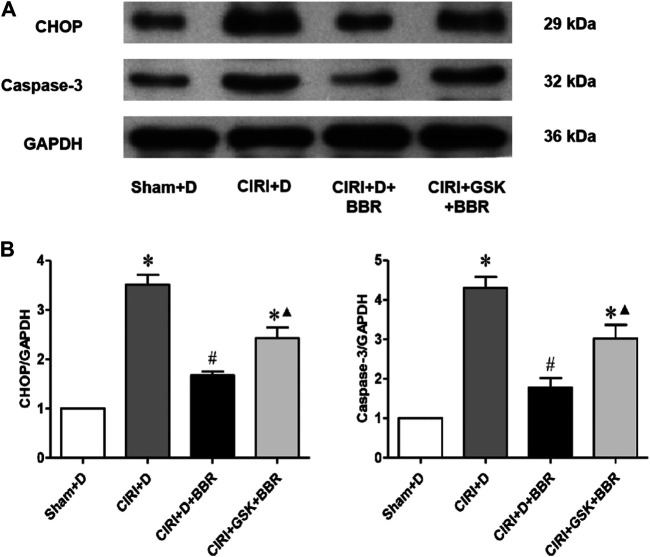
BBR attenuates neuronal apoptosis via the CNPY2-PERK pathway in cortical ischemic penumbra. The CNPY2-PERK apoptotic signaling proteins CHOP **(A)** and cleaved Caspase-3 **(B)** from cortical ischemic penumbra were determined by Western blot. The results were normalized to the percentage of GAPDH expression. **(B)**, Bar graph showing the statistical comparison of CHOP and cleaved Caspase-3 protein levels across groups. *n* = 6. Data are shown as the mean ± SEM. *p < 0.05 compared with the Sham + D group; ^#^ *p* < 0.05 compared with the CIRI group; ^▲^ *p* < 0.05 compared with the CIRI + D + BBR group.

To further confirm the effect of BBR on the CNPY2-PERK pathway induced by CIRI, we added a PERK-specific inhibitor to one of the treatment groups. Western blotting showed that compared with that in the CIRI + D + BBR group, the expression of CHOP and Caspase-3 in the CIRI + GSK + BBR group decreased significantly (*p* < 0.05, Figure 7). The experimental results confirmed that the BBR partially attenuates neuronal apoptosis after CIRI by inhibiting the CNPY2-PERK pathway.

## Discussion

Cerebrovascular diseases seriously endanger human health. In an aging population, the incidence of the disease will increase. Therefore, related basic and clinical research on ischemic stroke has become an area of intense active research. After ischemic stroke, blood perfusion can be rapidly restored by thrombolysis or mechanical recanalization (Burrows et al., 2015). However, some patients experience CIRI, which leads to further aggravation of neurological dysfunction (Chen et al., 2020). Our studies have shown that the CNPY2-PERK pathway plays an important role in CIRI, and BBR may exert neuroprotective effects by inhibiting this pathway. The current study reveals part of the mechanism by which BBR exerts a neuroprotective effect from the perspective of molecular biology and provides a theoretical basis for its clinical application in the treatment of ischemic stroke.

To improve the prognosis and quality of life of patients with ischemic stroke and to address various problems in the treatment of this condition, research has focused on the mechanism of neuronal damage after ischemia and the prevention of neuronal cell damage after recanalization. When cerebral ischemia occurs, the ischemic area can be divided into the ischemic core area and the ischemic penumbra according to histopathological characteristics (Uzdensky, 2019). The ischemic penumbra is the transition region between the ischemic core and the nonischemic area (Liu C. et al., 2018; Uzdensky, 2019). A large number of non-necrotic neurons are preserved in the ischemic penumbra owing to the small amount of collateral arteries. Their cell function is not completely destroyed and the damage can be reversed (Uzdensky, 2019). The key treatment for ischemic stroke is to restore the blood supply to the cerebral ischemic area as soon as possible and save the dying brain nerve cells (El Amki and Wegener, 2017). The cerebral tissue damage after CIRI, especially in the penumbra, plays an important role in determining the size of infarction and the prognosis of the stroke. Apoptosis strongly contributes to nerve cell death after CIRI and aggravates the damaged nerve function in patients with ischemic stroke (Uzdensky, 2019). In the first few hours after the stroke occurred in rodents, the expression levels of various apoptotic proteins were significantly upregulated in the ischemic area. At 0.5–1 h after MCAO, Caspase-3, Caspase-6 and Caspase-9 were overexpressed in the ischemic core of the cerebral cortex and further increased over the following 12–24 h (Ferrer et al., 2003; Krupinski et al., 2003). In the ischemic penumbra, Caspase-3 and Caspase-6 upregulation was detected in neurons 1–4 hours after stroke (Demyanenko and Uzdensky, 2017). Cytochrome C could be detected in the cerebral ischemic core area and in penumbra neuron cytoplasm 3 h after MCAO (Li et al., 2002; Ferrer and Planas, 2003). These findings indicate that apoptosis plays a key role in the pathological process of CIRI. The current study also suggested that CIRI increased the apoptosis of nerve cells, thereby increasing neurological dysfunction in rats.

In addition to the direct damage caused by ischemia and hypoxia, a variety of secondary injury mechanisms induced by ischemia/reperfusion aggravate neurological dysfunction, including excitatory amino acid toxicity, free radicals, inflammatory reactions, calcium overload, apoptosis and other pathological injury mechanisms (Datta et al., 2020). The interaction of various factors can be sequential or parallel and can affect the entire process of pathological development. Apoptosis is the main form of CIRI (Uzdensky, 2019). Current research suggests that there are three main signal transduction pathways that regulate apoptosis, namely, the mitochondrial pathway, the death receptor pathway and the ERS pathway (Ten and Galkin, 2019; Datta et al., 2020). The ERS-mediated apoptosis pathway plays an important role in the pathogenesis of CIRI (Xin et al., 2014). The ER is a membranous organelle and is the main site of calcium storage and signal transduction (Ron and Walter, 2007). The ER molecular chaperone protein GRP78/BIP (glucose regulatory protein 78/immunoglobulin heavy chain binding protein) is a marker protein of ERS (Gardner et al., 2013). Under physiological conditions, GRP78 forms stable complexes with three kinds of ER proteins: double-stranded RNA-activated protein kinase-like ER kinase (PERK), inositol-dependent factor 1 (IREl), and active transcription factor 6 (ATF-6). However, sustained and severe CIRI induces GRP78 to dissociate from these three transmembrane proteins, activates the PERK, IRE1, and ATF6 pathways, and transfers downstream stress signals to induce apoptosis (Degracia et al., 2002). This apoptosis pathway is mainly caused by C/EBP homologous protein (CHOP), apoptosis signal-regulated kinase 1 (ASK1) and Caspase-12 (Ferri and Kroemer, 2001; Iurlaro and Munoz-Pinedo, 2016). The expression of ERS apoptotic proteins (GRP78, p-PERK/PERK, CHOP and Caspase-3) in ischemic penumbra were found to be significantly increased after CIRI, and the expression levels of ERS apoptotic proteins (GRP78 and p-PERK/PERK) were further increased after the application of ER agonists. The experimental results confirmed that CIRI induces the ERS apoptosis pathway, thus aggravating the apoptosis of nerve cells.

As the main organ of signal transduction, the ER has a complex monitoring system and ensures the accuracy of signal transduction through a variety of regulatory mechanisms (Binet and Sapieha, 2015). Severe ischemia-reperfusion injury promotes the aggregation of unfolded or misfolded proteins in the ER cavity, disrupts the homeostasis of ER function, and thus determines cell survival (Luchetti et al., 2017). However, it is not completely clear how the ERS sensor is triggered by molecules. Recent studies have found that CNPY2 may be a promoter of ERS and participate in the occurrence of ERS (Hong et al., 2017). CNPY2 belongs to the canopy family and is widely present in various rat tissues, including in the nervous system, cardiovascular system, respiratory system, digestive system and reproductive system (Hatta et al., 2014; Guo et al., 2015b). During the transition of the ER from a nonstressed state to a stressed state, the CNPY2 binding partner switched from GRP78 to PERK and selectively activated the PERK-CHOP-mediated apoptosis signaling pathway (Urra and Hetz, 2017). CNPY2-deficient cells have also been produced by gene knockout in some studies, and PERK activation was found to be almost completely inhibited, indicating that CNPY2 expression is a key step in signal initiation (Hong et al., 2017). CNPY2 attenuated the transition from compensatory hypertrophy to ventricular dilation and heart failure (Guo et al., 2015a). Recent studies have found that barbaloin reduces myocardial ischemia-reperfusion injury in rats by inhibiting the CNPY2-PERK pathway (Cui et al., 2019). Our previous research also found that the CNPY2-ERS apoptosis pathway participated in the process of spinal cord ischemia-reperfusion injury (Zhao et al., 2019). However, as yet, the role of CNPY2 is poorly understood. The expression of CNPY2 in ischemic penumbra was found to be increased after CIRI and was further increased after the application of ER agonists. The experimental results suggest that CNPY2 is involved in the occurrence and development of CIRI. To further verify the role of the CNPY2-PERK pathway in CIRI, we added a PERK-specific inhibitor. The experimental results showed that the expression of ERS apoptotic proteins (CHOP and Caspase-3) in the ischemic penumbra significantly decreased. Combined with the above results, this indicated that CIRI activates the CNPY2-PERK apoptosis pathway and promotes the occurrence of apoptosis.

Coptis chinensis is a plant in the genus coptidis in the Ranunculaceae family and is a medicinal material used in China. Coptis chinensis contains a variety of alkaloids, and BBR is its main active ingredient. This alkaloid has a variety of pharmacological effects and is widely used clinically as an antipyretic analgesic and as an antibacterial agent for intestinal infections. In the past 20 years, in-depth studies on the pharmacological mechanisms of BBR have shown this compound to have antioxidant, antistress, anti-inflammatory, hypolipidemic and anticancer pharmacological activities (Wang et al., 2017). In addition, many clinical trials have clarified its therapeutic role in cardiovascular diseases, such as atherosclerosis, myocardial ischemia, heart failure and arrhythmia (Cicero and Baggioni, 2016). Excitingly, BBR is a natural medicine that can penetrate the blood-brain barrier and be absorbed by neurons. BBR is transmitted to the neurons through the blood-brain barrier in a concentration-dependent and time-dependent manner (Lin and Zhang, 2018). It has neuroprotective effects against a variety of central nervous system-related diseases, such as cerebral ischemia, Alzheimer's disease, schizophrenia, anxiety, and depression (Kulkarni and Dhir, 2010; Cicero and Baggioni, 2016). Recent studies have found that BBR plays a neuroprotective role after cerebral ischemia-reperfusion through a complex signaling network composed of multiple kinases and signaling pathways (Lin and Zhang, 2018). BBR was also found to promote angiogenesis in CIRI rats by activating the hypoxia-induced growth factor-1α (HIF-1α)/vascular endothelial growth factor (VEGF) signaling pathway (Liu H. et al., 2018). BBR can improve CIRI-induced injury by activating the brain-derived neurotrophic factor (BDNF)-promyosin receptor kinase B (TrkB)-PI3K/Akt signaling pathway (Yang et al., 2018). BBR may also play a neuroprotective role in the process of cerebral ischemia by blocking neuron channels in the hippocampal CA1 area, maintaining intracellular ion homeostasis and inhibiting apoptosis (Wang et al., 2004). These studies indicate that BBR has a significant neuroprotective effect against cerebral ischemic injury. However, the study of BBR from the perspective of ERS remains insufficient, and its potential molecular mechanism of protection is unclear. The current study found that BBR improved neurological dysfunction caused by CIRI and inhibited nerve cell apoptosis. Further molecular biology experiments found that BBR significantly inhibited CNPY2 expression and nuclear transfer and inhibited the expression of downstream apoptotic proteins (GRP78, p-PERK/PERK, CHOP and Caspase-3) in the CNPY2-ERS pathway. Additional experiments showed that BBR significantly inhibited the expression of CNPY2, GRP78, and p-PERK/PERK induced by ERS agonists. However, the inhibitory effect of BBR on proapoptotic proteins (CHOP and Caspase-3) was partially reversed after the application of PERK inhibitor. These experimental results indicate that BBR at least partially reduces the apoptosis induced by CIRI by inhibiting the CNPY2-PERK apoptosis pathway.

**FIGURE 8:**
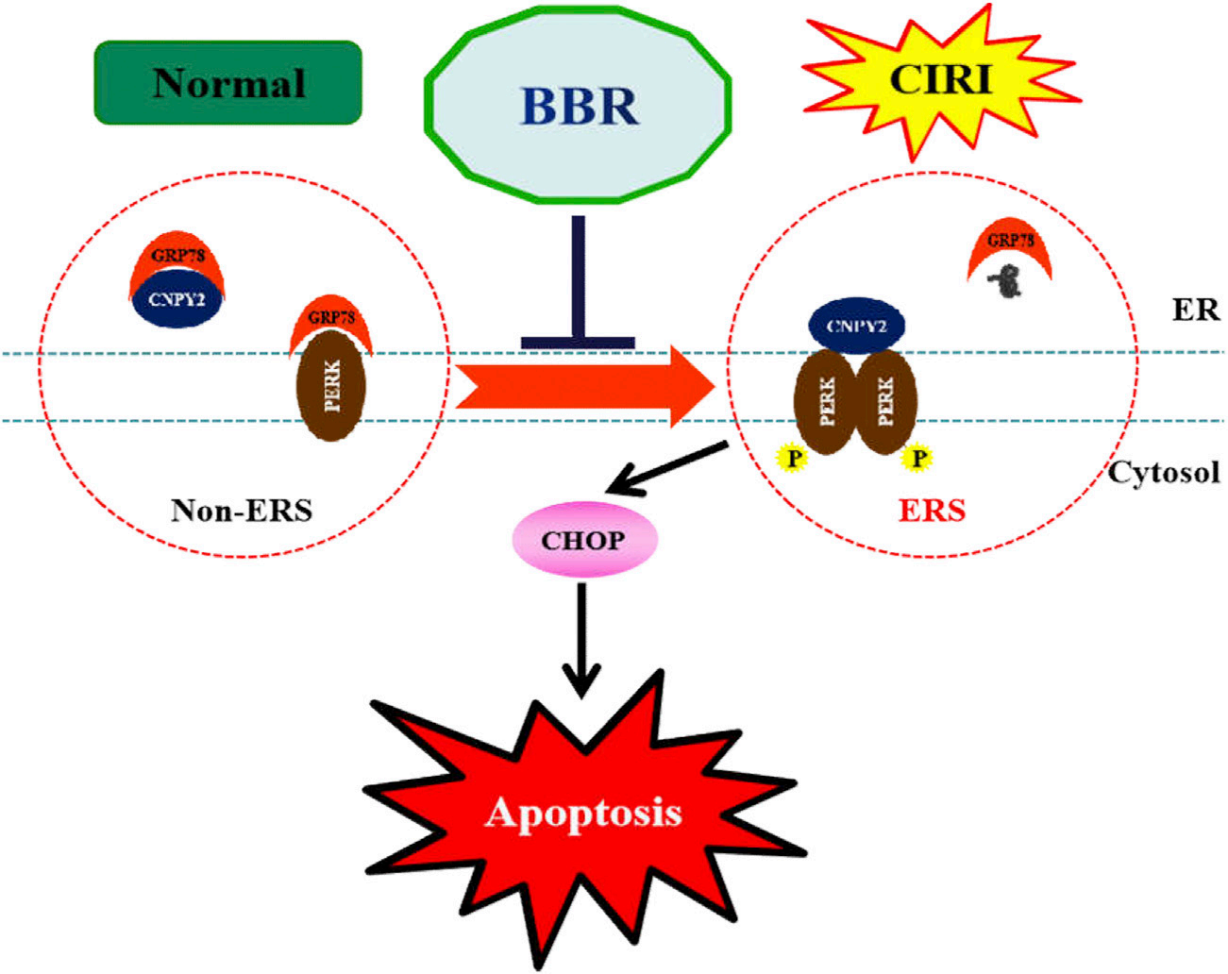
BBR alleviates ERS after CIRI. Berberine significantly inhibited CIRI induced up-regulation of CNPY2, GRP78 and CHOP, hallmarks of ERS. BBR, Berberine; CIRI, cerebral ischemia-reperfusion injury; CNPY2, canopy FGF signaling regulator 2; GRP78: glucose-regulated protein 78; CHOP: C/EBP homologous protein.

In summary, this study showed the protective effect of BBR against CIRI in rats and further identified the important role of the CNPY2-ERS pathway in the neuroprotective mechanism of BBR (Figure 8). These results reveal part of the neuroprotective mechanism of BBR and provide a theoretical basis for the clinical application of BBR in ischemic stroke. However, this study also has limitations. Whether BBR may play a protective role under different conditions of CIRI conditions is still unknown. Similarly, it is not clear whether BBR can reduce CIRI through ERS-related inflammation, autophagy or other signaling pathways, which is also worthy of further exploration. Therefore, we will continue to study the possible protective mechanism of BBR on CIRI to accelerate the rehabilitation of patients, so as to fully develop the potential advantages and good development prospects of traditional Chinese medicine.

## Data Availability

The raw data supporting the conclusion of this article will be made available by the authors, without undue reservation.
