# Supplementary figures and images for: Berberine Attenuates Cerebral Ischemia-Reperfusion Injury Induced Neuronal Apoptosis by Down-Regulating the CNPY2 Signaling Pathway
Source: Front Pharmacol. 2021 Apr 28;12:609693. doi: 10.3389/fphar.2021.609693 (PMC8113774; doi:10.3389/fphar.2021.609693)

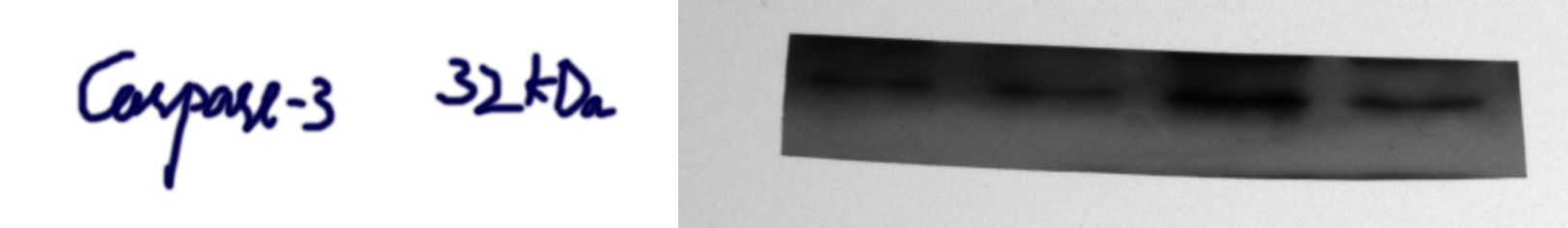

Supplement: Supplementary file 1 [file datasheet1.zip › Supplementary images/Fig. 2C Caspase-3.tif]

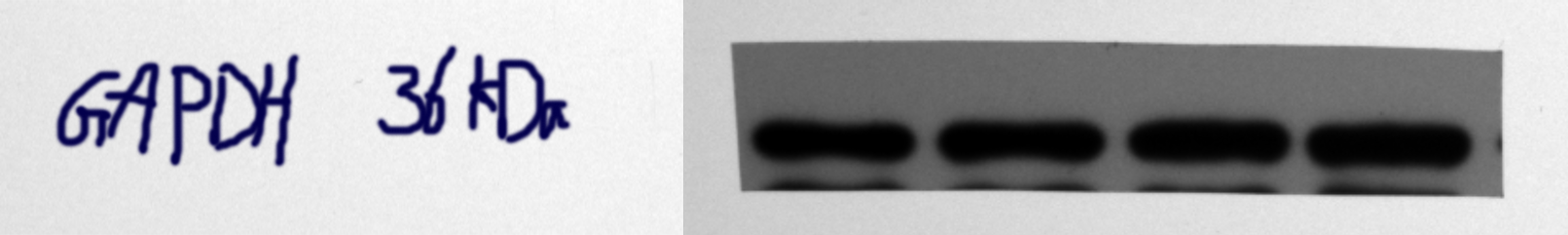

Supplement: Supplementary file 1 [file datasheet1.zip › Supplementary images/Fig. 2C GAPDH.tif]

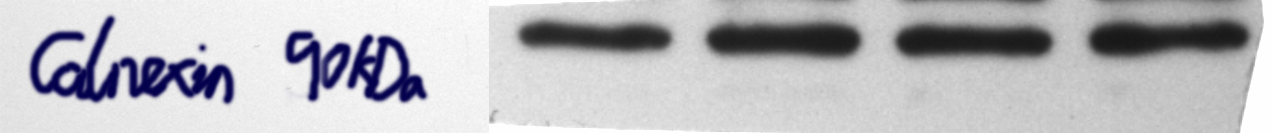

Supplement: Supplementary file 1 [file datasheet1.zip › Supplementary images/Fig. 3A Calnexin.tif]

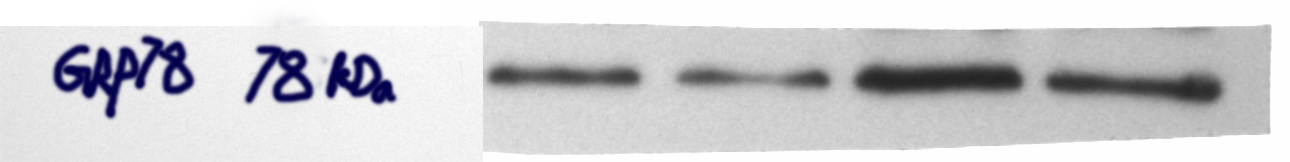

Supplement: Supplementary file 1 [file datasheet1.zip › Supplementary images/Fig. 3A GRP78.tif]

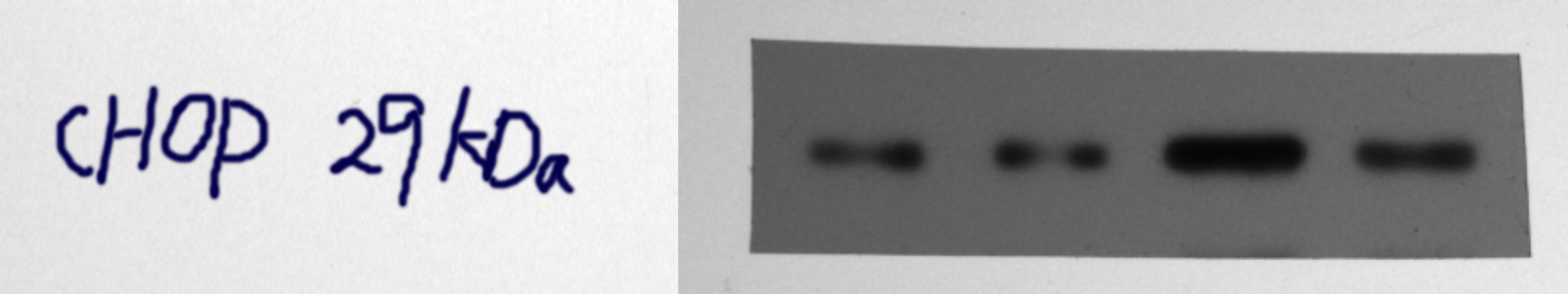

Supplement: Supplementary file 1 [file datasheet1.zip › Supplementary images/Fig. 3B CHOP.tif]

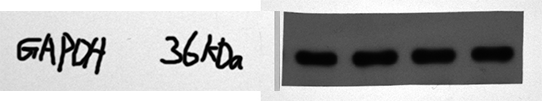

Supplement: Supplementary file 1 [file datasheet1.zip › Supplementary images/Fig. 3B GAPDH.tif]

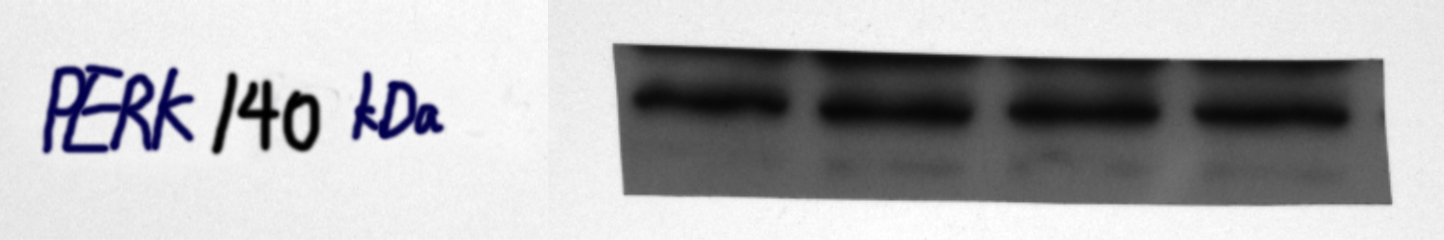

Supplement: Supplementary file 1 [file datasheet1.zip › Supplementary images/Fig. 3B PERK.tif]

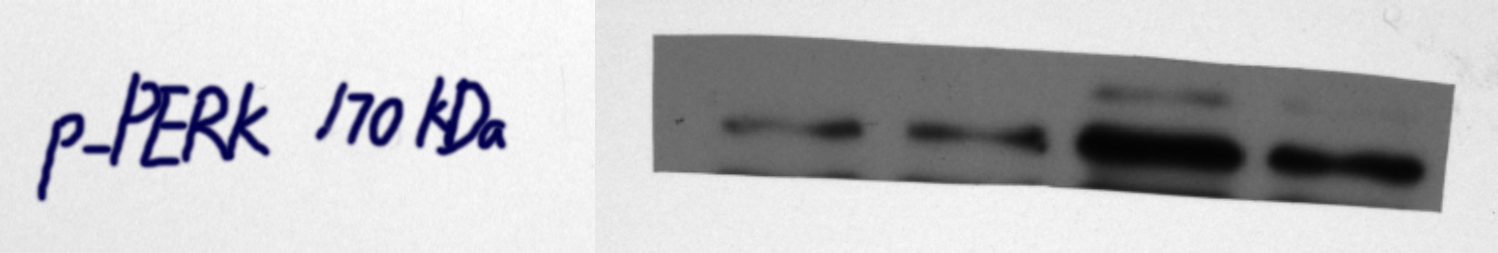

Supplement: Supplementary file 1 [file datasheet1.zip › Supplementary images/Fig. 3B p-PERK.tif]

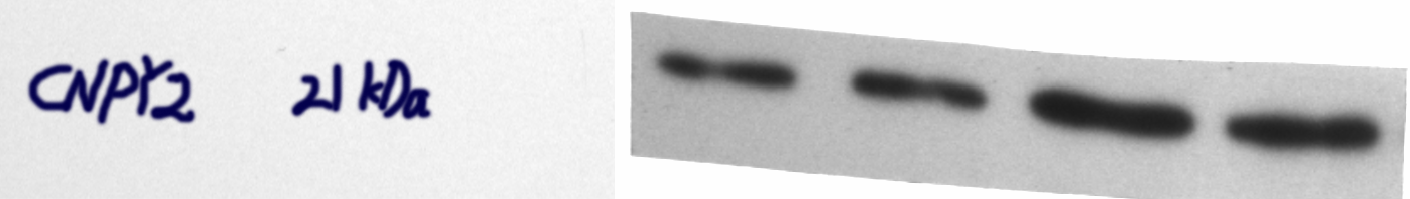

Supplement: Supplementary file 1 [file datasheet1.zip › Supplementary images/Fig. 6A CNPY2.tif]

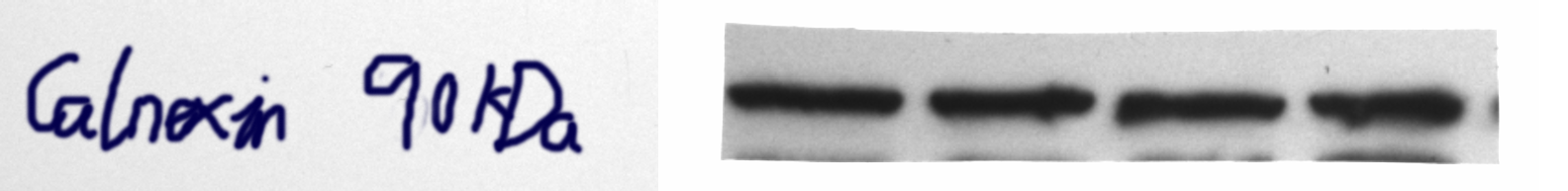

Supplement: Supplementary file 1 [file datasheet1.zip › Supplementary images/Fig. 6A Calnexin.tif]

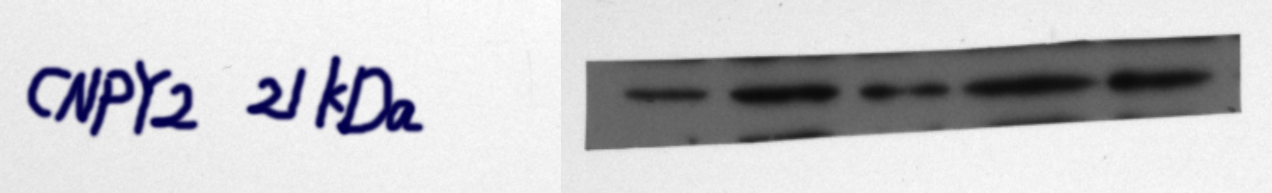

Supplement: Supplementary file 1 [file datasheet1.zip › Supplementary images/Fig. 6B CNPY2.tif]

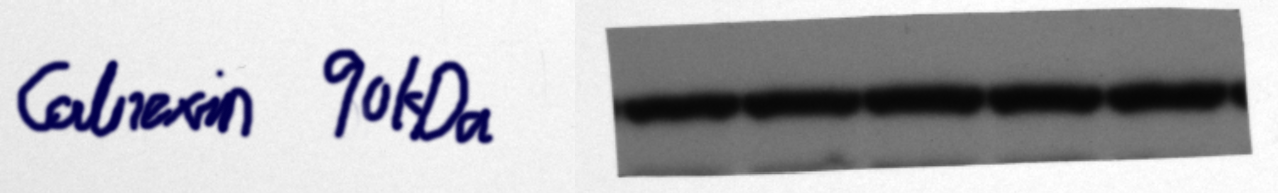

Supplement: Supplementary file 1 [file datasheet1.zip › Supplementary images/Fig. 6B Calnexin.tif]

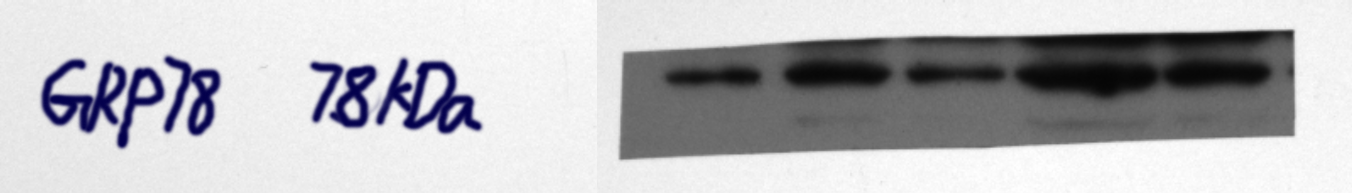

Supplement: Supplementary file 1 [file datasheet1.zip › Supplementary images/Fig. 6B GRP78.tif]

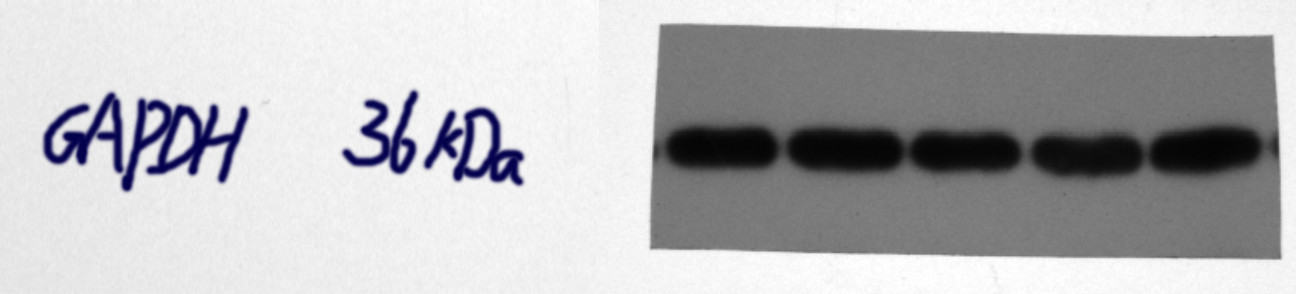

Supplement: Supplementary file 1 [file datasheet1.zip › Supplementary images/Fig. 6D GAPDH.tif]

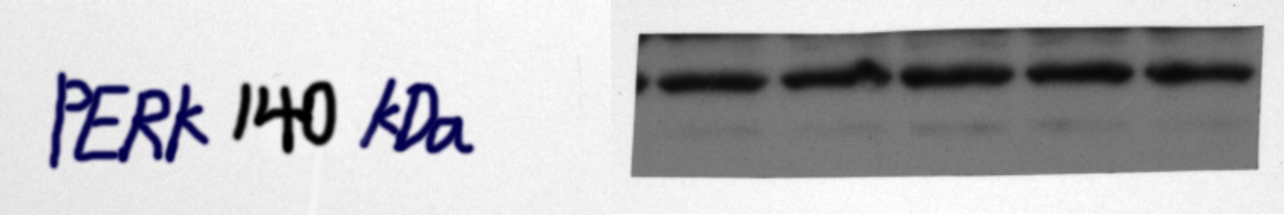

Supplement: Supplementary file 1 [file datasheet1.zip › Supplementary images/Fig. 6D PERK.tif]

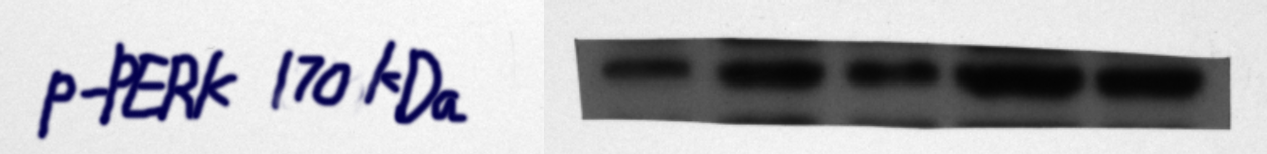

Supplement: Supplementary file 1 [file datasheet1.zip › Supplementary images/Fig. 6D p-PERK.tif]

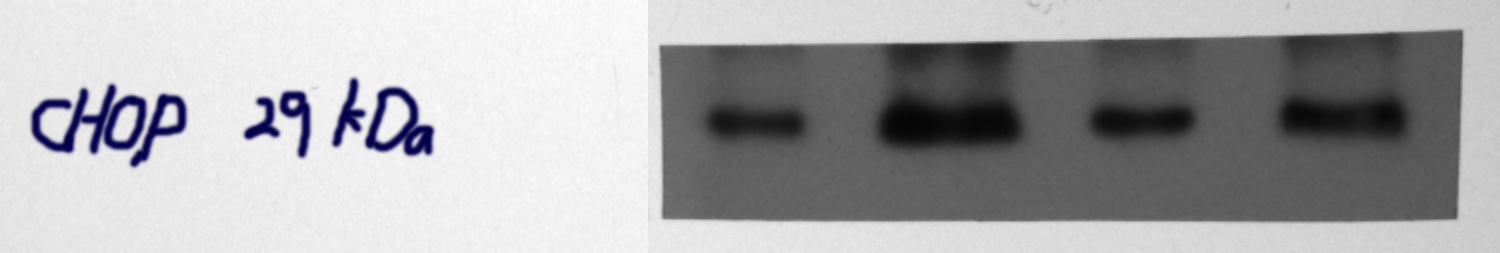

Supplement: Supplementary file 1 [file datasheet1.zip › Supplementary images/Fig. 7A CHOP.tif]

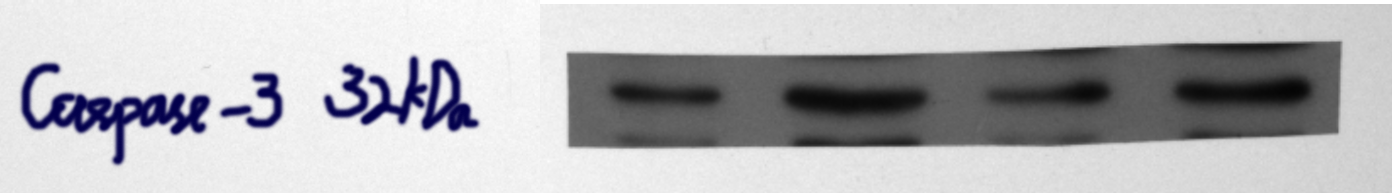

Supplement: Supplementary file 1 [file datasheet1.zip › Supplementary images/Fig. 7A Caspase-3.tif]

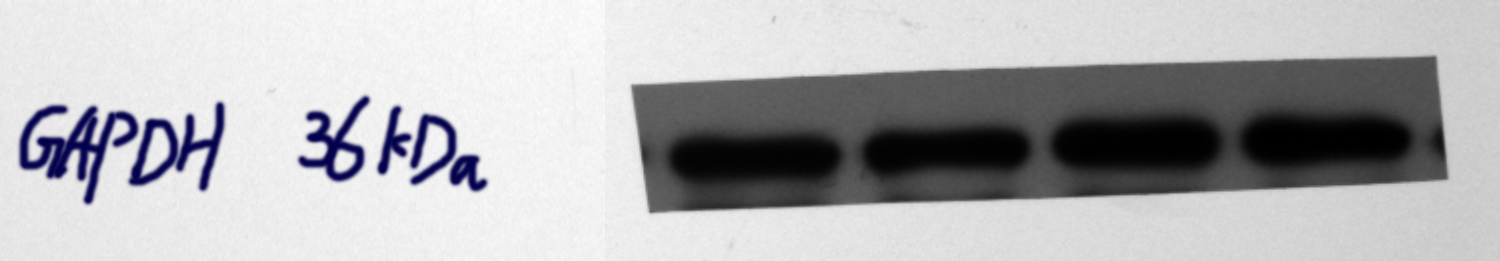

Supplement: Supplementary file 1 [file datasheet1.zip › Supplementary images/Fig. 7A GAPDH.tif]
